# The Examination of Horses for Soundness1Read before the Veterinary Medical Association of New York County, March 3, 1897.

**Published:** 1897-05

**Authors:** H. D. Hanson

**Affiliations:** New York City


### THE EXAMINATION OF HORSES FOR SOUNDNESS.[1]

By H. D. Hanson, D.V.S.,
NEW YORK CITY.

The subject of the examination for soundness is not a new one to you, but, as it is very interesting as well as important, I have selected it in order to try to excite discussion and bring out some of the important points that I have only touched upon or have omitted. My paper is short, being only a rough outline of the subject, as time will not permit a lengthy discourse.

In my opinion, the subject of the examination for soundness ought to be considered by veterinarians as follows: First, theoretical soundness; second, practical soundness.

Theoretical soundness rarely, if ever, exists, which should be remembered in making our examinations. This being the case, we, as veterinarians, have to be contented, and are compelled to make use of the term "practical soundness," which should be defined to be that condition existing which is the nearest approach to a perfectly sound horse.

[1] Read before the Veterinary Medical Association of New York County, March 3, 1897.

Before proceeding any further, it is well to understand the meaning of "soundness." In defining a sound horse it becomes necessary to combine in a few words that which is difficult or almost impossible, as a complete definition and explanation would consume many pages.

Suffice it to say that a sound horse is one that is free from all disease and has nothing about him that interferes or is likely to interfere with his usefulness, or depreciate his value as a sound horse.

Taking the above definitions and the conditions really existing in practice, what do we find, and how are we to proceed?

In the first place, we find that theoretical soundness rarely, if ever, exists; second, that practical soundness is what we have to deal with; third, that a horse must be free from all disease that renders him unable to do his kind of work as a sound horse should; fourth, that certain conditions may be present which, in a longer or shorter time, are likely to render him unfit to do the work of a sound horse; fifth, that nothing must exist that tends to depreciate the value of the animal as compared to a sound horse; sixth, that we, as veterinarians, ought to examine carefully and conscientiously, taking into consideration the class of horse (whether a smooth-limbed, well-bred animal, or whether a common-bred, rough, and big-boned animal), as well as the kind of work the horse will have to do; seventh, that we, as veterinarians, have our reputation at stake; eighth, that we must do our duty to the buyer who employs us; ninth, that we should not be prejudiced against the seller; tenth, that in acting fairly for ourselves we will do our duty to all concerned.

How are we to proceed to examine the animal? We must bear in mind that a horse may be temporarily unsound and permanently unsound.

Temporary unsoundness may include certain diseases, of which, after the animal has recovered, little or no trace remains; it also includes certain forms of lameness, as interfering, slight sprains, etc., which subside in a short while. In our examinations we should look for disease, malformations, etc., and not for soundness.

The horse to be examined should be seen, first, at rest, in and out of the stall; second, in motion, both when warmed up and when cooled off (that is, examined in motion while hot and also when cold).

While at rest in the stall we may ascertain how the animal feeds —if he cribs, sucks wind, weaves, his position of standing, etc.; when backing out of the stall, the movements, whether there is dragging of the feet, or other peculiar actions.

On the floor, have the animal naked (nothing on but a halter). Now take a general glance at the animal, noting the position of the extremities, the color, size, and general outline. Then proceed to examine part by part, commencing at the head and not forgetting to compare one side with the other as you go along.

In examining the head note the shape, the conditions of the bones of the face, the shape and condition of the eyes, the pupils, the ears, the mucous membranes of the nasal cavity (looking at the color, for ulceration, growths, etc.); then examine the contents of the mouth, the age, the shape, size, and wearing surfaces of the teeth, and examine the bars for fractures; next pass to the inter-maxillary space, feeling the pulse, the condition of the lower jaw, whether thickened or thinned; the glands in this region, then the poll, the throat, the condition of the glands, not forgetting to cause the animal to cough and remembering the kind of cough; passing along the neck, the condition of the mane, the jugular groove, the trachea; the shoulders for marks of setons, wasting of muscles; the elbows (for capped elbow); the knee for fractures, inflammation, etc.; the shin-bones for splints; the tendons (for their condition); the fetlock for fractures, swellings, scars of neurotomy, etc.; then look for side-bones, ring-bones, etc.; now the general outline of the feet, as regards size, shape, etc., and in particular for toe-cracks, quarter-cracks, results of laminitis, navicular disease, etc.

Next the body comes under our observation, remembering to examine both sides; first the withers, looking for marks of setons, swellings, etc.: then the condition of the ribs; auscultate the heart and lungs; then pandiculate, looking at the abdomen for hernia, etc.; the flank, noting the respirations, whether quickened, slow, irregular, and the like.

Next place yourself behind the animal, examining the hips for fractures, swellings, etc.; then the hip-joints (remembering to compare one side with the other); now examine the tail to see if false; look about the anus for tumors; the inguinal region for hernia, tumors, etc.; the stifles (for swellings, dislocations); the hocks (for spavins, thoroughpins, curbs); then pass downward, noting the condition of the parts as in the anterior extremities. While here do not forget to take the temperature.

Now have the animal trotted by the halter in as straight a line as possible in a slow, easy trot, allowing it about one foot of rope, so as not to interfere with the action; the animal should be trotted from you, at which time you note the movements of the posterior extremities, and when trotted toward you the movements of the anterior extremities.

It is also well to have the animal turned both ways, so as to see if any signs of stringhalt exist.

When the animal is warmed up lameness may not be shown, while if cooled off it manifests itself, and *vice versa*. Next have the animal galloped to test his wind; see if the breathing is fast, irregular, or noisy Do not forget to examine the eyes with the ophthalmoscope.

I have given you a rapid sketch of the examination, but there still remains another very important part, which is the writing of a certificate.

What ought a certificate contain? It ought to contain the age, sex, height, condition of the mane and tail, and any special peculiarities or marks that may exist, the latter identifying one horse of the same color from another. If an animal be unsound, the cause of lameness, etc., may be mentioned in the certificate.

The following is the usual form used and adopted by Prof. Liautard:

This is to certify that I have examined this day, at the request of Mr.          , a chestnut gelding, six years old, fifteen and a half hands high, clipped mane, full tail, white star on forehead, low white stocking on near foreleg, high white stocking on off hind, and find said animal to be lame on the near hindleg from a spavin, and therefore unsound, or have failed to find any unsoundness.

---

The foreign demand for American-bred horses continues to enlarge, and almost every great selling centre of our country has representatives of foreign countries buying for export. The enhancing values of good horses may in a measure limit the sale in this direction.

Two notable features of the recent carriage exhibition at the Philadelphia Bourse was the absence of any horseless vehicles and a specially designed carriage for Veterinarian Charles Williams, with an electric headlight for the top, self-oiling axles, and a medicine-drawer, with compartments, which slides out of sight under the seat. It follows somewhat in design the Jenny Lind style.

